# Reduced Expression of *PTPRD* Correlates with Poor Prognosis in Gastric Adenocarcinoma

**DOI:** 10.1371/journal.pone.0113754

**Published:** 2014-11-20

**Authors:** Dandan Wang, Leilei Wang, Jun Zhou, Jihong Pan, Wei Qian, Jiafang Fu, Genglin Zhang, Youming Zhu, Chunshan Liu, Chunliang Wang, Zongkun Jin, Ziqing He, Jianmei Wu, Bin Shi

**Affiliations:** 1 Key Laboratory for Biotech-Drugs Ministry of Health, Key Laboratory for Modern Medicine and Technology of Shandong Province, Key Laboratory for Rare & Uncommon Diseases of Shandong Province, Key Laboratory for Virology of Shandong Province, Back and Neck Pain Hospital of Shandong Academy of Medical Sciences, Shandong Medicinal Biotechnology Centre, Shandong Academy of Medical Sciences, Jinan, People's Republic of China; 2 Biology Institute of Shandong Academy of Sciences, Jinan, People's Republic of China; 3 Departments of Oncology, Provincial Hospital Affiliated to Shandong University, Jinan, People's Republic of China; 4 The General Hospital of Jinan Military Command, Jinan, People's Republic of China; The University of Hong Kong, China

## Abstract

**Background:**

*PTPRD*, encoding protein tyrosine phosphatases receptor type D, is located at chromosome 9p23–24.1, a loci frequently lost in many types of tumors. Recently, *PTPRD* has been proposed to function as a tumor suppressor gene. The current study aimed to investigate *PTPRD* expression and its prognostic significance in primary gastric adenocarcinoma.

**Methods and Results:**

Quantitative real time reverse transcription PCR (qRT-PCR) and western blotting were used to examine *PTPRD* expression in paired gastric tumourous and paracancerous tissues. Compared with the matched normal gastric mucosa tissues, both the mRNA (P = 0.0138) and protein (P = 0.0093) expression of *PTPRD* in fresh surgical specimens were significantly reduced. Clinicopathological and prognostic roles of *PTPRD* in gastric adenocarcinoma were investigated using immunohistochemistry with 513 paraffin-embedded gastric adenocarcinoma tissue blocks. Statistical analysis revealed that reduced *PTPRD* expression was significantly associated with T stage (P = 0.004), TNM stage (P<0.001) and tumor size (P = 0.003). Furthermore, Kaplan-Meier survival analysis revealed that low expression of *PTPRD* significantly correlated with poor survival of gastric cancer patients (P<0.001). Cox regression analysis confirmed *PTPRD* expression as independent predictor of the overall survival of gastric cancer patients. The MTT assay determined the effects of *PTPRD* on cell proliferation of MGC803 and GES1 cell lines. Restoring *PTPRD* expression in MGC803 cells significantly inhibited their growth rate. Silencing *PTPRD* expression by siRNA treatment in GES1 significantly enhanced cell proliferation compared with mock siRNA treatment. Methylation analysis of *PTPRD* promoter CpG island in 3 primary GC samples showed one case with partial methylation.

**Conclusions:**

These results indicated that *PTPRD* is a candidate tumour suppressor in gastric cancer. Thus, *PTPRD* may play an important role in gastric tumorigenesis and serve as a valuable prognostic marker of gastric adenocarcinoma.

## Introduction

Globally, gastric cancer (GC) is currently the fourth most common malignancy and the second leading cause of cancer mortality [1]. More new cases of GC are diagnosed in China each year than in any other country [2]. The incidence of GC has declined over time, due to improving living standards, improvements in early diagnosis, advanced surgical techniques and combined therapy (surgery, chemotherapy and radiotherapy) [3]. However, distant metastasis and local recurrence cannot be avoided easily in most cases, and the prognosis of GC patients remains far from satisfactory [2], [3]. Tumorigenesis of GC has been considered a multifactorial and multistep process that involves the activation of oncogenes and the inactivation of tumor suppressor genes at different stages [4]. Further understanding of these alterations and the molecular mechanisms involved in gastric carcinogenesis will be critical for improved diagnosis, therapy and prognosis of GC.

Protein tyrosine phosphatases (PTPs) are signaling molecules that regulate a variety of cellular processes, including cell growth, differentiation, cell cycle and oncogenic transformation. The constitutive activation of PTPs signaling pathways is a biochemical hallmark of cancer [5]. This is mostly occurs via activation of tyrosine kinase receptors, such as amplification of HER2/Neu and mutations of the epidermal growth factor receptor [5]. The protein encoded by the *PTPRD* gene (protein tyrosine phosphatase, receptor type, D) is one of 38 known human receptor-type PTPs, a group of proteins that are increasingly thought to be important in human neoplasia and cancer progression [6], [7].

The *PTPRD* gene is located at chromosome 9p23–24.1, a locus frequently lost in neuroblastoma, gliomas, lung cancer and other malignancies [8]–[11]. Weir et al. detected homozygous deletions and missense mutations of *PTPRD* in adenocarcinoma of the colon and lung [12], [13]. David et al. identified frequent deletion and mutation of *PTPRD* in glioblastoma multiforme and malignant melanoma, and showed that these mutations were inactivating [5]. A recent study showed reduced *PTPRD* expression in the majority (>80%) of cell lines and surgical specimens of lung cancer, indicating that *PTPRD* is a candidate tumor suppressor [14]. These researches suggested that *PTPRD* might be one of a select group of tumor suppressor genes that are inactivated in a wide range of common human tumor types.

However, the role of *PTPRD* in human gastric adenocarcinoma has not yet been investigated. In the present study, we detected *PTPRD* expression level in gastric adenocarcinoma using quantitative real-time reverse transcription PCR (qRT-PCR), western blotting and immunohistochemistry. Meanwhile, prognostic and clinicopathological features of *PTPRD* were investigated in 513 gastric adenocarcinoma tissue samples. Furthermore, we evaluated the functional role of *PTPRD* in the proliferation of the GC cell line MGC803 and gastric epithelial mucosa cell line GES1. We further designed methylation-specific PCR assays to assess the methylation status of *PTPRD* promoter CpG island in primary GC tissues. Taken together, our research suggested that *PTPRD* was a candidate tumor suppressor in GC. Low expression of *PTPRD* was a reliable indicator of disease progression and poor prognosis of GC.

## Materials and Methods

### Ethics Statement

The research was approved by the Ethics Committee of Shandong Academy of Medical Sciences. Written informed consent was obtained from each patient involved in the study.

### Cell line and culture conditions

The GC cell line MGC803 and gastric epithelial mucosa cell line GES1 were obtained from the Committee of Type Culture Collection of Chinese Academy of Sciences (Shanghai, China). The cells were cultured in RPMI 1640 media containing 10% heat-inactive fetal bovine serum (FBS). The cells were incubated at 37°C in a humidified 5% CO_2_ atmosphere.

### Human tissue samples

A total of 42 paired fresh GC specimens and matched adjacent noncancerous tissue samples were collected from GC patients undergoing radical gastrectomy at Sun Yat-sen University Cancer Center between 2010 and 2012, and the diagnosis was confirmed by pathological examination. None of the patients had been treated before sugery. After surgical resection, fresh tissues were immediately immerged in RNA later (Ambion, Inc., USA) to avoid RNA degradation, and then stored at 4°C overnight to allow thorough penetration of RNA later into the tissue. Next, all the samples were frozen at −80°C until RNA and protein extraction was performed. Another 3 fresh GC specimens, used for methylation analysis of *PTPRD*, were collected from GC patients undergoing radical gastrectomy at Sun Yat-sen University Cancer Center in September 2014. The tumor-Node-Metastasis (TNM) staging was recorded based on the 7th edition of the International Union Against Cancer (UICC).

### Gastric cancer patients and follow-up

Five hundred and thirteen paraffin-embedded primary gastric carcinoma samples were obtained from the postoperative patients in Sun Yat-sen University Cancer Center between January 2003 and December 2006. All patients in our study belonged to the same ethnic group. The patients were selected according to the criteria: (1) diagnosis of gastric adenocarcinoma with histopathological identification; (2) limited or extended surgical history that included gastrectomy plus lymphadenectomy; (3) no chemotherapy and radiotherapy before surgery; (4) availability of complete follow-up data; (5) no history of other synchronous malignancies or familial malignancy; (6) no recurrent gastric cancer or remnant gastric cancer; (7) no death in the perioperative period. The surgery was performed by experienced surgeons followed procedures of Japanese Gastric Cancer Association (JGCA) guidelines.

Postoperative follow-up included clinical and laboratory examinations every 3 months for the first 2 years, every 6 months during the third to fifth years, annually for an additional 5 years or until patient death, whichever occurred first. The overall survival was defined as the time from the operation to the death or last follow-up. The characteristics of these patients are listed in table 1.

**Table 1:** Correlation between *PTPRD* expression and clinicopathological parameters of 513 gastric adenocarcinoma cases.

Clinicopathological parameters	*n* a	*PTPRD* expression		χ^2^	*P* value
		High	Low		
**All**	513	252	261		
**Age (years)**					
<55	230	117	113	2.713	0.257
≧55	281	133	148		
**Gender**				0.037	0.853
Male	338	165	173		
Female	175	87	88		
Tumor size				9.241	0.003*
<3 cm	84	54	30		
≧3 cm	429	198	231		
**Tumor infiltration**				15.182	0.004*
T1	67	42	25		
T2	53	34	19		
T3	175	86	89		
T4a	179	73	106		
T4b	39	17	22		
**Local lymph node metastasis**				4.237	0.238
N0	189	103	86		
N1	119	58	61		
N2	82	38	44		
N3	123	53	70		
**Distant metastasis**				2.426	0.128
M0	466	234	232		
M1	47	18	29		
**TNM staging**				20.785	<0.001*
1	95	64	31		
2	192	98	94		
3	177	71	106		
4	49	19	30		

a ^a^Numbers of cases in each group. * Statistically significant (*P*<0.05).

### Extraction of total RNA and qRT-PCR

Total RNA was extracted using TRIzol (Invitrogen, Carlsbad, California, USA) according to the protocol of manufacturer's. RNAse-free DNase I was used to remove DNA contamination. Total RNA concentration was assessed by measuring absorbance at 260 nm using a NANO DROP spectrophotometer (ND-1000, Thermo Scientific, USA). Reverse transcription (RT) to synthesize the first-strand of cDNA was performed in a 25 µl reaction volume containing 2 µg total RNA, 0.5 µg Oligo (dt), 200 U M-MLV reverse transcriptase (Promega, USA), 25 U RNase inhibitor and 2.5 mM dNTP. The reaction system was incubated at 70°C for 5 minutes, 42°C for1 hour and resulting cDNA was stored at −20°C. The cDNA was then subjected to real-time quantitative PCR for evaluation of the relative mRNA levels of *PTPRD* and *GAPDH* (glyceraldehyde-3-phosphate dehydrogenase, as an internal control) with the following primers: *PTPRD* forward: 5′-TTATCAGTGCCAATCTTC-3′, and reverse: 5′-TCTGTTGTCTGTATCCAT-3′; *GAPDH* forward: 5′-CTCCTCCTGTTCGACAGTCAGC-3′, and reverse: 5′-CCCAATACGACCAAATCCGTT-3′. Gene-specific amplification was performed using an ABI 7900HT real-time PCR system (Life Technologies, Carlsbad, California, USA) with a 15 µl PCR reaction system containing 0.5 µl cDNA, 7.5 µl of 2 x SYBR Green master mix (Invitrogen, Carlsbad, California, USA), and 200 nM of the appropriate oligo nucleotide primers. The reaction procedure was performed as follows: preheated at 95°C for 10 min, and then 45 cycles of amplified at 95°C for 30 secands 60°C for 1 min. The resolution curve was measured at 95°C for 15 sec, 60°C for 15 sec and 95°C for 15 sec. The Ct (threshold cycle) value of each sample was calculated from the threshold cycles with the instrument's software (SDS 2.3). Relative expression levels of *PTPRD* were normalized to the geometric mean of the internal control gene *GAPDH*. Data were analyzed using the comparative threshold cycle (2^−ΔCT^) method.

### Western blotting analysis

The frozen gastric cancer tissue samples, including tumor and non-tumor tissues, as well as cell lines, were lysed in RIPA lysis buffer at 4°C for 15 min. The lysates were cleared by centrifugation (12,000 rpm) at 4°C for 30 min to collect total protein. About 50 µg protein samples were then separated by electrophoresis in a 12% SDS (sodium dodecyl sulfate) polyacrylamide gel and the transferred onto a polyvinylidene fluoride membrane. After blocking the non-specific binding sites for 60 min with 5% non-fat milk, the membranes were incubated with a rabbit polyclonal antibody against *PTPRD* (LifeSpan BioScineces, USA, at 1∶1000 dilution) at 4°C overnight. The membranes were then washed with TBST (tris-buffered saline with tween-20) three times at room temperature for 15 min. After washing, the target protein was probed with the horseradish peroxidase (HRP)-conjugated goat anti-rabbit IgG antibody (SantaCruz, USA, at 1∶2000 dilution) at 37°C for 1 hour. After three washes, the membranes were developed by an enhanced chemiluminescence system (Cell Signaling Technology, Danvers, Massachusetts, USA). The band intensity was measured by densitometry using the Quantity One software (Bio-Rad Laboratories, Inc. Hercules, CA, USA). The protein levels were normalized with respect to *GAPDH* protein level which was detected using mouse anti-human *GAPDH* monoclonal antibody (Shanghai Kangchen, China, at 1∶5000 dilution).

### Immunohistochemistry analysis

After deparaffinization with dimethylbenzene, the gastric cancer tissue sections were rehydrated through 100%, 95%, 90%, 80% and 70% ethanol. After three washes in PBS (phosphate-buffered saline), the tissue sections were boiled in antigen retrieval buffer containing 0.01 M sodium citrate-hydrochloric acid (pH = 6.0) for 15 min using a microwave oven. After rinsing with PBS, the sections were incubated with primary antibody and then rinsed in 3% peroxidase quenching solution (Invitrogen) to block endogenous peroxidase. The sections were then incubated with a rabbit polyclonal antibody against *PTPRD* (LifeSpan BioScineces, USA, at 1∶100 dilution) at 4°C overnight. After washing with PBS, the sections were incubated with a biotinylated secondary antibody (Zhongshan Golden Bridge Biotech, Beijing, China) at room temperature for 30 min. The visualization signal of the slides was treated with 3, 3′-diaminobenzidine (DAB) solution, and all of the slides were counterstained with hematoxylin for 15 min. As negative controls, adjacent sections were processed as described above except incubating at 4°C overnight in blocking solution without the primary antibody.

### Semi-quantitative evaluation

The *PTPRD* protein expression level was assessed by immunostaining score, which was calculated as the sum of the percent of positively stained tumor cells and the staining intensity. Briefly, the percentage of positive staining was scored as 0 (0–9%, negative), 1 (10%–25%, sporadic), 2 (26%–50%, focal) or 3 (51%–100%, diffuse), and the intensity as 0 (no staining), 1 (weak staining, visible at high magnification), 2 (moderate staining, visible at low magnification) and 3 (dark staining, strikingly positive at low magnification). The total immunostaining score was calculated with the value of percent positivity score × staining intensity score, which ranged from 0 to 9. The expression level of *PTPRD* was defined as following: “−” (negative, score 0), “+” (weakly positive, score 1–3), “++” (positive, score 4–6), and “+++” (strongly positive, score7–9). Based on the *PTPRD* expression levels, we divided the gastric cancer patients into two groups: low *PTPRD* expression group (*PTPRD* “−” or *PTPRD* “+”) and high *PTPRD* expression group (*PTPRD* “++”or *PTPRD* “+++”).

### Expression plasmid and transient transfections

A eukaryotic expression plasmid pCMV6-XL4 containing the full-length of human *PTPRD* cDNA was obtained from the ORIGENE Company (Beijing, China). Empty vector was used as negative control. MGC803 cells (2×10^5^) were cultured in 6-well plates until they reached 85–90% confluence, and then transient transfections were performed using Lipofectamine 2000 (Invitrogen) according to the manufacturer's protocol. At 48 hours after transfection, gene expression was examined by qRT-PCR and western blotting analysis. After that, cell proliferation assay was performed.

### RNA oligonucleotides and cell transfection

For knockdown of *PTPRD* expression, the siRNAs were synthesized by GenePharma Company (Shanghai, China). The siRNA sequences were as follows: siRNA1-*PTPRD*, forward: 5′-GAGCCACACAGAAGUUAAUUU-3′, reverse: 5′-AUUAACUUCUGUGUGGCUCUU-3′, and siRNA2-*PTPRD*, forward: 5′-GUGGGUCUGUUAUCAUUAUTT-3′, reverse: 5′-AUAAUGAUAACAGACCCACTT-3′. The negative control (NC), forward: 5′-UUCUCCGAACGUGUCACGUTT-3′, reverse: 5′-ACGUGACACGUUCGGAGAATT-3′. 400 pmol siRNA-*PTPRD* or NC was transfected into 2×10^5^ GES1 cells using Lipofectamine RNAi MAX reagent (Invitrogen, USA) for 48 hours according to the manufacturer's protocol. After detecting by qRT-PCR and western blotting, cell proliferation assay was performed.

### Cell proliferation assay

Cell growth rate was detected by MTS assay. Cells were seeded in a 96-well plate at a density of 3×10^2^ cells per well. The cell growth rate was detected using cell proliferation MTS kit according to the manufacturer's instruction (Promega, USA). Each experiment was performed in triplicate.

### Methylation analysis

The genomic DNA of 3 fresh GC tissues was isolated with the DNeasy Tissue Kit (Qiagen Inc., Valencia, CA). Bisulfite modification of genomic DNA was carried out using the CpGenome DNA Modification Kit (Chemicon). Bisulfite modified DNA was amplified by PCR using two sets of *PTPRD* specific primer pairs that recognize either the methylated or unmethylated CpG island and then analyzed by electrophoresis. Selection of primers used for methylation-specific PCR was accomplished using MSPPrimer [15]. Primer sequences are as follows: M-*PTPRD* (methylated), forward: 5′-GGGGTTCGTTTAGGTCGC-3′, reverse: 5′-CGCCCGCTAAAAAAAAAAACGACG-3′, and U-*PTPRD* (unmethylated), forward: 5′-TGGTGGGGTTTGTTTAGGTTGTG-3′, reverse: 5′-ATACTCCAAACACCCACTAAAAAAAAAAACAACA-3′.

### Statistical analysis

Differences in *PTPRD* mRNA and protein expression between paired tumor and the adjacent non-tumor tissue samples were evaluated with the paired Student's *t*-test. The *χ^2^*test was used to analyze the relationships between *PTPRD* expression and various clinicopathological parameters. Survival curves were calculated using the Kaplan–Meier method and compared by the log-rank test. Univariate and multivariate analyses were performed to detect *PTPRD* expression and the clinicopathological variables by using Cox proportional hazards regression model. The two-tailed unpaired Student's *t*-test was used to assess differences in cell growth rate. All the statistical analyses were performed with the software of SPSS (Statistical Package for the Social Sciences, version 17.0, Chicago, IL, USA), and a two-sided P value less than 0.05 was considered to be statistically significant.

## Results

### qRT-PCR analysis of *PTPRD* mRNA expression

**Figure 1:**
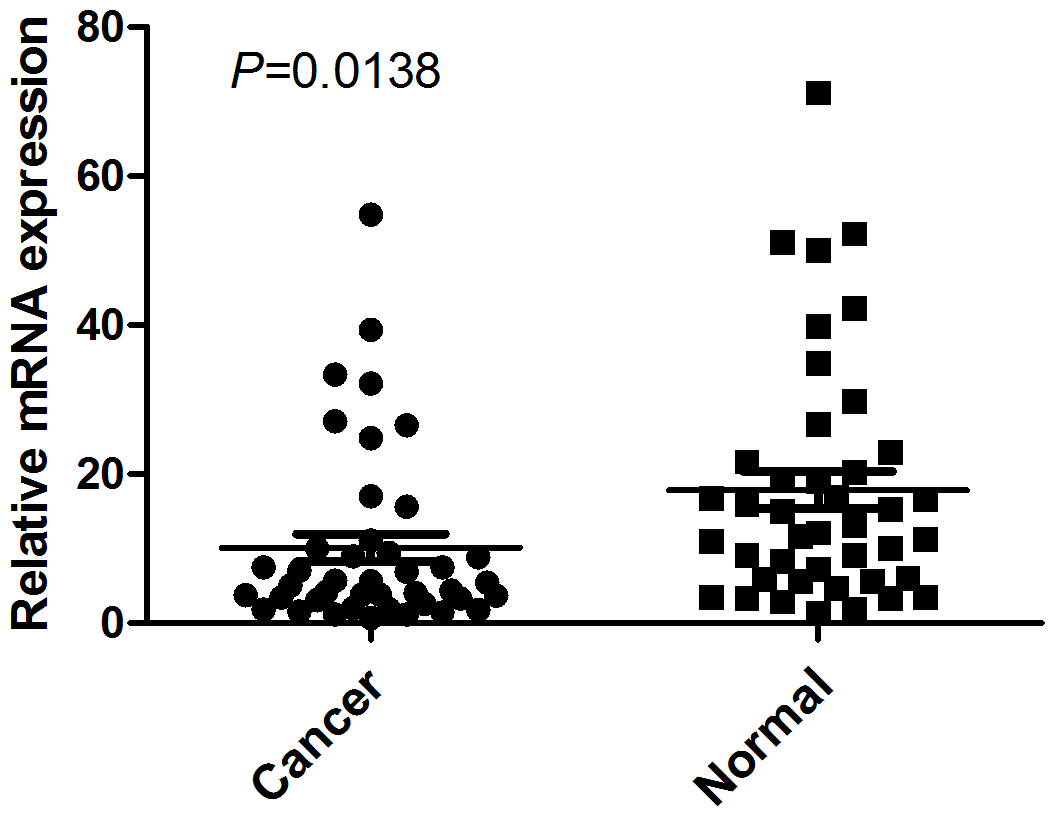
The mRNA expression of *PTPRD* in human primary gastric adenocarcinoma surgical specimens was evaluated by qRT-PCR. The relative mRNA expression of *PTPRD* was significantly decreased in GC tissues compared with the matched adjacent noncancerous tissues (n = 42, P = 0.0138). Horizontal lines represent the mean.

The mRNA level of *PTPRD* was examined by qRT-PCR assays in 42 paired gastric cancerous and matched adjacent normal mucosa tissues. As shown in figure 1, the *PTPRD* expression level was significantly lower in 32 (76.19%) tumor-bearing tissues compared with the adjacent non-tumor tissues (P = 0.0138, Figure 1).

### Western blotting analysis of *PTPRD* protein expression

**Figure 2:**
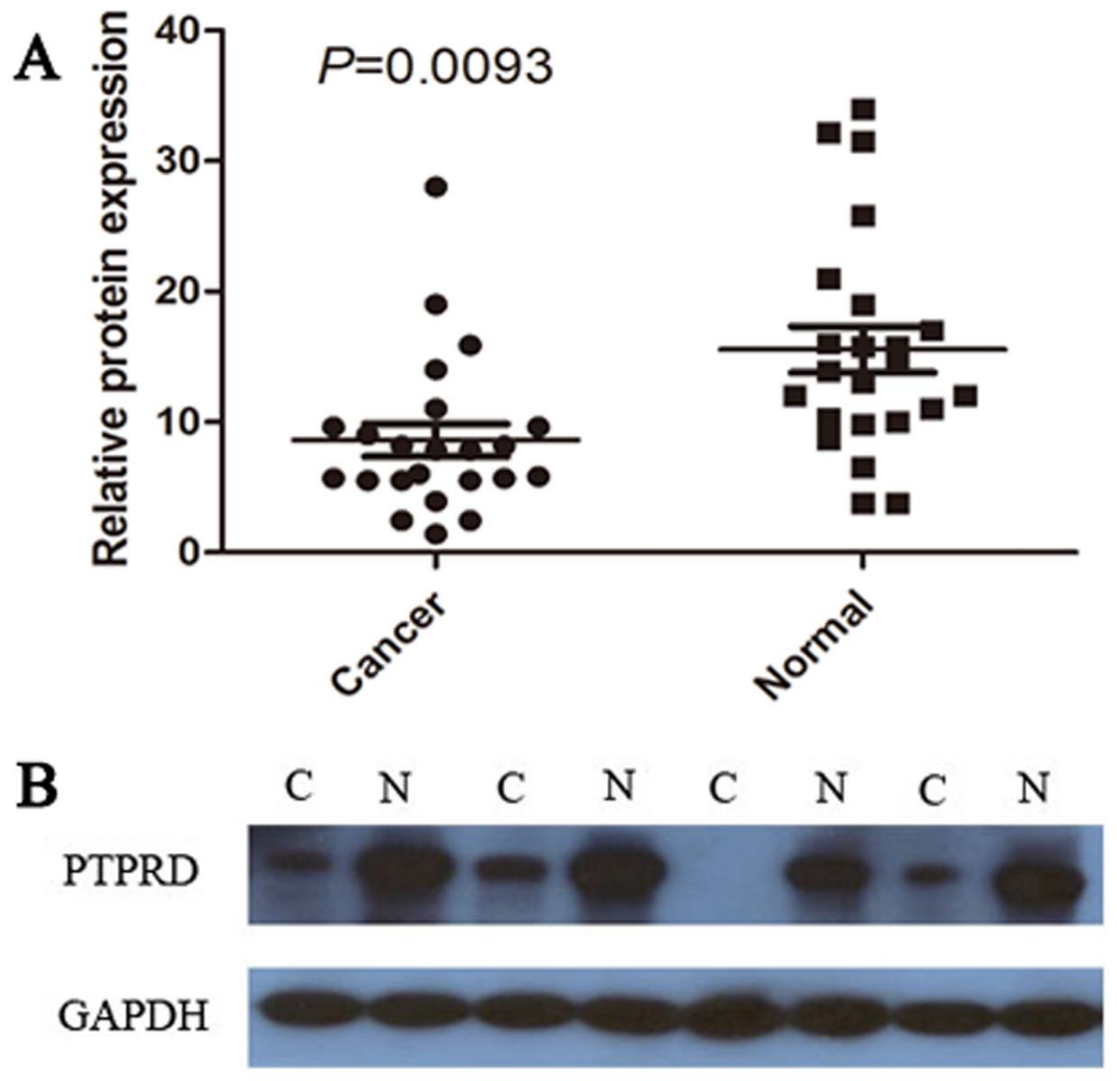
Decreased protein expression of *PTPRD* in gastric adenocarcinoma was assessed by western blotting. (A) Relative *PTPRD* protein expression levels in GC tissues and the matched normal paracancerous tissues (*PTPRD*/*GAPDH*, n = 23, P = 0.0093). Horizontal lines represent the mean. (B) Representative results of *PTPRD* protein expression in 4 paired GC tissues and the matched adjacent noncancerous tissues (C, GC tissues; N, matched noncancerous gastric mucosa).

To further investigate if the expression of *PTPRD* is reduced at the protein level in gastric adenocarcinoma, western blotting was performed on 23 paired gastric cancerous and noncancerous specimens. The results showed a *PTPRD* band at the expected size of 250 kDa, and the amount of *PTPRD* protein present was further measured by densitometry (normalized to *GAPDH* expression as a loading control). Fifteen of 23 (65.22%) tumors showed decreased *PTPRD* expression. The statistical evaluation showed significantly decreased expression of *PTPRD* in gastric tumor tissues compared with matched adjacent noncancerous tissues (P = 0.0093, Figure 2A). Representative examples of western blotting results were shown in Figure 2B.

### Immunohistochemical analysis and clinicopathological characteristics

**Figure 3:**
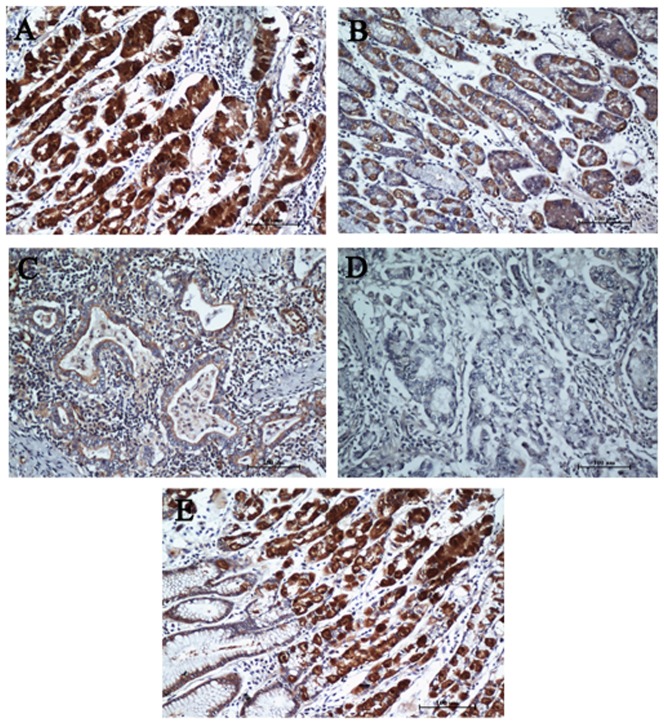
*PTPRD* protein expression in gastric adenocarcinoma surgical specimens evaluated by immunohistochemistry. (A) Strong *PTPRD* staining was observed in noncancerous gastric mucosa. (B) Strong *PTPRD* staining in well-differentiated gastric cancer. (C) Weak *PTPRD* staining in moderately differentiated GC. (D) Negative *PTPRD* staining in poorly differentiated GC. (E) Immunostaining of GC and adjacent nontumorous tissues showing a sharp contrast of *PTPRD* staining intensity.

To further investigate the clinicopathological and prognostic roles of *PTPRD* expression, 513 paraffin-embedded gastric adenocarcinoma tissue blocks were used for immunohistochemical analysis. Results indicated that 261 of 513 (50.87%) cases showed reduced *PTPRD* expression in cancerous tissues (Figure 3C&D), whereas 252 (49.12%) cases showed strong immunostaining (Figure 3B). Normal gastric mucosa showed the strongest *PTPRD* positive staining (Figure 3A). Immunostaining of a gastric cancer sample from the same patient showed a sharp contrast of *PTPRD* staining intensity (Figure 3E). In addition, low expression of *PTPRD* was significantly correlated with tumor size (P = 0.003), depth of tumor infiltration (T stage, P = 0.004), and TNM stage (P<0.001), but not with age, gender, local lymph node metastasis (N stage), or distant metastasis (M stage) (Table 1). Representative photomicrographs are shown in Figure 3.

### Expression of *PTPRD* and clinical outcome

**Figure 4:**
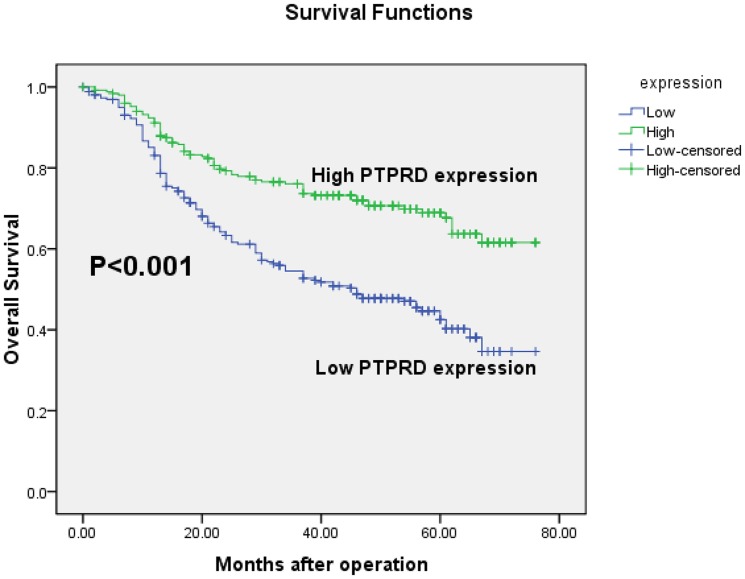
Kaplan-Meier survival curves of gastric adenocarcinoma patients (n = 513) after gastrectomy. The survival rate of patients in *PTPRD*-high group was significantly higher than that of the patients in the *PTPRD*-low group (log-rank test, P<0.001).

The prognostic value of *PTPRD* for gastric adenocarcinoma patients' overall survival was evaluated between patients with high and low *PTPRD* protein levels. The 5-year overall survival rates in patients with high and low *PTPRD* expression were 67.7% and 42.5%, respectively. Kaplan-Meier curve assessment showed that the overall survival of patients with low *PTPRD* expression was significantly worse than that of *PTPRD*-high patients (P<0.001, log-rank test, Figure 4).

To identify the potentially significant prognostic variables in all the patients with gastric adenocarcinoma, univariate Cox regression analysis of each variable was performed in relation to the survival time. Data showed that tumor infiltration, local lymph node metastasis, distant metastasis, TNM stage, tumor size and *PTPRD* expression were significantly associated with overall survival (Table 2). Furthermore, multivariate Cox regression analysis confirmed local lymph node metastasis (P = 0.011), TNM stage (P = 0.038) and *PTPRD* expression (P = 0.002) as independent predictors of overall survival of GC patients (Table 2). The relative risk of death in patients with high-*PTPRD* tumors was significantly lower than that of patients with low-*PTPRD* tumors (HR = 0.629).

**Table 2:** Univariate and multivariate analyses of overall survival of gastric adenocarcinoma patients.

Variables	*n* ^a^	Univariate analyses			Multivariate analyses		
		HR	(95% CI)	*P* value	HR	(95% CI)	*P* value
**Age (years)**				0.332			
<55	230	1.000					
≧55	281	1.139	0.875–1.483				
**Gender**				0.719			
Female	175	1.000					
Male	338	0.951	0.723–1.251				
**Tumor size**				<0.001*			0.690
<3 cm	84	1.000			1.000		
≧3 cm	429	4.257	2.321–7.810		1.144	0.591–2.213	
Tumor infiltration				<0.001*			0.167
T1	67	1.000			1.000		
T2	53	7.847E3	0.000–8.853E29		1.075E4	0.000–4.118E37	
T3	175	3.322E4	0.000–3.734E30		1.416E4	0.000–5.437E37	
T4a	179	3.914E4	0.000–4.400E30		1.299E4	0.000–4.987E37	
T4b	39	7.998E4	0.000–8.997E30		2.229E4	0.000–8.566E37	
Local lymph node metastasis				<0.001*			0.011*
N0	189	1.000			1.000		
N1	119	2.477	1.608–3.817		1.644	1.020–2.651	
N2	82	4.022	2.605–6.209		1.915	1.118–3.281	
N3	123	5.931	3.996–8.803		2.416	1.435–4.068	
Distant metastasis				<0.001*			0.600
M0	466	1.000			1.000		
M1	47	6.330	4.479–8.946		0.673	0.153–2.957	
TNM staging				<0.001*			0.038*
1	95	1.000			1.000		
2	192	10.615	3.329–33.849		2.414	0.552–10.551	
3	177	26.111	8.293–82.210		3.211	0.673–15.306	
4	49	91.390	28.270–295.440		18.176	2.174–151.950	
*PTPRD*				<0.001*			0.002*
Low	261	1.000			1.000		
High	252	0.474	0.357–0.630		0.629	0.469–0.845	

HR, hazard ratio; CI, confidence interval; ^a^ Numbers of cases in each group; * Statistically significant (*P*<0.05).

### The role of *PTPRD* in cell proliferation in MGC803 and GES1 cell lines

**Figure 5:**
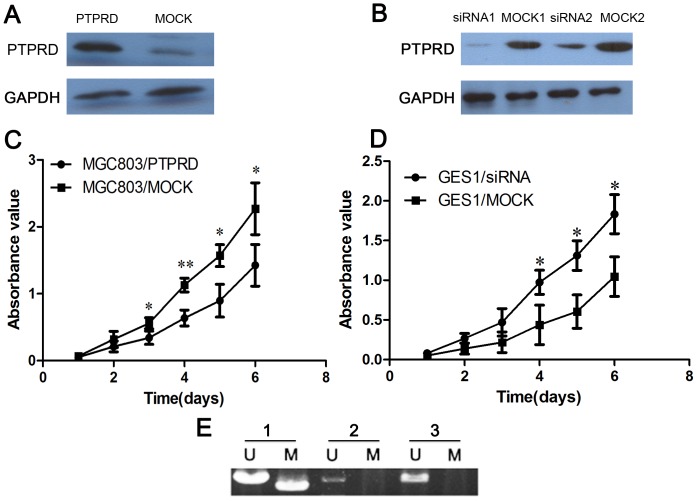
The growth suppressor role of *PTPRD* in cell proliferation and DNA methylation analysis of *PTPRD*. (A) Western blotting analysis of *PTPRD* overexpression in MGC803 cells. (B) Western blotting analysis of decreased *PTPRD* expression in GES1 cells. (C) Cell proliferation assay showing the suppressive effect of restoring *PTPRD* expression on the proliferation of MGC803 cell line. (D) Results showing significantly enhanced proliferation rate of *PTPRD*-silenced GES1 cells compared with mock siRNA treatment GES1 cells. (E) Methylation analysis of *PTPRD* promoter CpG island in primary GC tissues. Among 3 GC samples, one case showed partial methylation and 2 cases were unmethylated. *, P<0.05 versus the mock control; **, P<0.01 versus the mock control.

To further evaluate the effects of *PTPRD* on cell proliferation, a *PTPRD* expression vector and control vector were transfected separately into MGC803 cells. *PTPRD* expression in transfected cells were detected by western blotting (Figure 5A). Cell growth assay revealed that the cell growth rate in *PTPRD*-transfected GC cells was significantly lower than that of control vector-transfected GC cells (Figure 5C). Meanwhile, we silenced *PTPRD* expression in the gastric epithelial mucosa cell line GES1 using *PTPRD*-specific siRNA oligonucleotides. The *PTPRD* expression in transfected GES1 cells were detected by western blotting (Figure 5B). Silencing the expression of *PTPRD* in GES1 significantly enhanced cell proliferation compared with mock siRNA treatment (Figure 5D).

### Abnormal DNA methylation of *PTPRD* in GC

We further designed methylation-specific PCR assays to assess the methylation status of *PTPRD* promoter CpG island in primary GC tissues. Results showed that, among 3 GC samples, one case showed partial methylation and 2 cases were unmethylated (Figure 5E).

## Discussion

Receptor protein tyrosine phosphatase delta (*PTPRD*) is a member of the highly conserved family of receptor protein tyrosine phosphatases (PTPs) [15]. The PTPs are a superfamily of enzymes that function in a coordinated manner with protein tyrosine kinases to control signalling pathways that underlie a broad spectrum of fundamental physiological processes [7]. These enzymes are divided into the classical group, phosphotyrosine (pTyr)-specific phosphatases and the dual specificity phosphatases [7], [16], [17]. There are 107 PTPs encoded in the human genome, of which 38 belong to the group of classical PTPs, which show specificity for phosphotyrosine [6]. PTPs are signaling molecules that regulate a variety of cellular processes, including cell growth, differentiation, mitotic cycle and oncogenic transformation [6], [7]. Recently, several classical PTPs have been identified as potential tumor suppressors, including receptor PTPs such as DEP1 (densityenhanced phosphatase-1, encoded by *PTPRJ*) [18], PTPκ (encoded by *PTPRK*) [19] and PTPρ (encoded by *PTPRT*) [20]. This group of genes is increasingly thought to be important in cancer development and progression.

The *PTPRD* gene is located at chromosome 9p23–24.1, an area of human genome that is frequently lost in many kinds of tumors [8]–[11]. Urushibara et al. described a selective reduction in *PTPRD* expression in hepatomas and first proposed *PTPRD* as a tumor suppressor [21]. Subsequent studies reported homozygous deletions of *PTPRD* in a broad spectrum of human tumor types, such as lung adenocarcinoma [9], [12], [22], [23], pancreatic carcinoma [24], melanoma [25] and glioblastoma [5], etc. Kohno et al. observed reduced *PTPRD* expression in the majority (>80%) of cell lines and surgical specimens of lung cancer [14]. Veeriah et al. found that *PTPRD* was mutated in 6% of glioblastoma multiformes, 13% of head and neck squamous cell carcinomas, and in 9% of lung cancers [15]. Their study revealed that loss of expression of *PTPRD* predicts for poor prognosis in glioma patients [15]. These studies have established that *PTPRD* has a growth suppressive role in many types of human cancer [26]. However, thus far, the expression, clinical significance and biological functions of *PTPRD* in gastric adenocarcinoma have not been explored.

In our present study, we detected the mRNA and protein levels of *PTPRD* in GC patients by western blotting and qRT-PCR, respectively. *PTPRD* was expressed at both lower mRNA and protein levels in GC tissues compared with corresponding non-cancerous tissues. Moreover, immunohistochemistry showed decreased *PTPRD* expression in 261 out of 513 samples of gastric cancer patients. These results indicated that *PTPRD* might be a candidate tumour suppressor in GC. Our observation is in agreement with a series studies revealing that *PTPRD* expression is frequently lost or reduced in a number of human cancer tissues and cell lines, including lung cancer and glioblastoma multiforme [14], [15].

The correlation of *PTPRD* and clinical outcome was analyzed by immunohistochemical staining of specimens in large series of gastric cancer patients (n = 513). Reduced expression of *PTPRD* was significantly correlated with tumor size (P = 0.003), depth of tumor infiltration (T stage, P = 0.004), and TNM stage (P<0.001). The overall survival of patients with low *PTPRD* expression was significantly worse than that of *PTPRD*-high patients (P<0.001). These findings were similar to the previous studies in lung cancer and glioblastoma by Veeriah et al. [15]. Taken together, these results demonstrated that *PTPRD* might serve as a tumor suppressor in a broad spectrum of human tumor types.

Univariate and multivariate analysis demonstrated that *PTPRD* was an independent risk factor in the prognosis of GC patients. Thus, *PTPRD* may serve as a valuable prognostic biomarker for GC patients after surgery and as a potential target for gene therapy in the treatment of GC.

*PTPRD* encodes a transmembrane protein with a cytoplasmic tyrosine phosphatase domain [15]. Recently, a study by Veeriah et al. revealed that loss of *PTPRD* resulted in altered growth of astrocytes [15]. *PTPRD* directly dephosphorylates the oncoprotein STAT3 and regulates the STAT3 pathway. Mutations in *PTPRD* abrogate the ability to regulate STAT3 [15]. Their results suggest that *PTPRD* may act as a tumor suppressor by regulating cell growth, and the loss of this gene plays an important role in progression, rather than the initiation of malignant gliomas [15]. In the current study, we found that the loss of *PTPRD* expression was significantly correlated with a higher T stage of gastric cancer, implying that absence of *PTPRD* expression may promote tumor growth and invasion. Moreover, we detected lower *PTPRD* immunoreactivity in poorly differentiated gastric cancer tissues than in well-differentiated ones, suggesting that decreased *PTPRD* expression might play a role in tumor de-differentiation. Furthermore, we investigated the functional role of *PTPRD* in MGC803 and GES1 cell lines. Restoring *PTPRD* expression in GC cells significantly inhibited cell proliferation. Whereas, silencing *PTPRD* expression in gastric epithelial cells significantly enhanced the cell growth rate. These results indicated that *PTPRD* might play an import role in regulating gastric cancer cell growth. Recently, Veeriah's research showed that human astrocytes lacking *PTPRD* exhibited increased growth [15]. Our study, together with that of Veeriah et al., suggested that *PTPRD* might serve as a candidate tumour suppressor in a wide range of common human tumor types. However, the functional role and molecular underpinnings of *PTPRD* in GC have not been fully explored, requiring further investigation in future research.

DNA hypermethylation in promoter CpG island has been shown to be a predominant mechanism by which tumor suppressors are inactivated in cancers [27]. In the present study, methylation analysis of *PTPRD* promoter CpG island in 3 primary GC samples showed one case with partial methylation. Low *PTPRD* expression in most GC tissues was probably due to DNA methylation of promoter CpG island. Recently, two studies showed DNA hypermethylation of *PTPRD* in glioblastoma and breast cancer cell lines [15], [28]. These researches indicated that the methylation of CpG in the *PTPRD* promoter was might be involved in the inactivation of *PTPRD* in many types of human cancers.

In conclusion, we demonstrated reduced *PTPRD* expression in gastric adenocarcinoma and its correlation with a more malignant phenotype and poorer prognosis in a large number of clinical samples. In addition, the data generated in the current study represent a valuable report correlating the presence of *PTPRD* with clinicopathological characteristics and the overall survival of gastric cancer patients. Further studies are needed to fully evaluate the molecular mechanism of low expression of *PTPRD* in gastric oncogenesis. We confirmed that *PTPRD* might serve as a candidate tumor suppressor gene and prognostic biomarker in gastric adenocarcinoma.
